# Irradiated Human Umbilical Vein Endothelial Cells Undergo Endothelial-Mesenchymal Transition via the Snail/miR-199a-5p Axis to Promote the Differentiation of Fibroblasts into Myofibroblasts

**DOI:** 10.1155/2018/4135806

**Published:** 2018-02-12

**Authors:** Minxiao Yi, Bo Liu, Yang Tang, Fang Li, Wan Qin, Xianglin Yuan

**Affiliations:** Department of Oncology, Tongji Hospital, Huazhong University of Science and Technology, Wuhan, Hubei Province, China

## Abstract

Radiation induced pulmonary fibrosis (RIPF) is one of the major side effects of radiotherapy for lung cancer. Previous studies have shown that endothelial cells and activated myofibroblasts play a key role in RIPF. However, the interaction between irradiated endothelial cells and activation of myofibroblasts has not been reported. The aim of the present study was to examine whether irradiated endothelial cells would affect the differentiation of fibroblasts into myofibroblasts in the process of RIPF. In the current study, we used a coculture system that allowed direct contact between human fetal lung fibroblasts (MRC-5) and irradiated human umbilical vein endothelial cells (HUVECs). After 24 or 48 h, cells were sorted by flow cytometry. Radiation induced endothelial-mesenchymal transition (EndMT) by significantly increasing the expression of Snail and vimentin and reducing the expression of CD31 in HUVECs. In addition, irradiation of HUVECs induced the expression of collagen type I and *α*-smooth muscle actin (*α*-SMA) in MRC-5 cells. Further investigation indicated that irradiation of HUVECs induced the differentiation of fibroblasts into myofibroblasts through the Snail/miR-199a-5p axis. We conclude that irradiated endothelial cells undergo EndMT to promote differentiation of fibroblasts into myofibroblasts via the Snail/miR-199a-5p axis.

## 1. Introduction

Approximately 60% of patients with non-small-cell lung cancer receive radiation therapy [1]. However, the dose for radiation therapy is often limited by the side effects of radiation induced lung injury (RILI) [2]. RILI mainly includes pneumonitis and pulmonary fibrosis, and ordinarily RILI specifically refers to radiation induced pulmonary fibrosis (RIPF) [3]. Although RIPF can affect the quality of life of patients and even lead to fatal respiratory insufficiency, there is no effective treatment for RIPF [4].

RIPF is characterized by excessive extracellular matrix (ECM) deposition, which occurs 6 to 12 months after lung irradiation. In pulmonary fibrosis, activated myofibroblasts play a crucial role in the production of collagen and ECM proteins [5]. A large number of studies have shown that the main source of myofibroblasts is resident fibroblasts [6]. The activation of fibroblasts is affected by a variety of cells such as epithelial cells, endothelial cells, and various factors such as CTGF, TGF*β*, and endothelin [7, 8].

A study has demonstrated that injury first occurs in endothelial cells within a few weeks after irradiation, before the formation of pulmonary fibrosis [9]. In the early stages of RIPF, collagen deposition induced by irradiation occurs mainly around the blood vessels, which becomes apparent throughout the irradiated tissue [10]. During this process, endothelial cells undergo endothelial-mesenchymal transition (EndMT), which is characterized by loss of the endothelial cell phenotype such as CD31 expression and acquisition of an interstitial cell phenotype such as vimentin expression [11].

There is growing evidence that the transcriptional activator Snail is an important regulator of EndMT in fibrosis [12]. Studies have shown that Snail-induced EndMT can promote myofibroblast activation in the development of cardiac fibrosis [13]. In RIPF, endothelial cells undergo EndMT and thus directly differentiate into myofibroblasts, thereby promoting the occurrence of pulmonary fibrosis [10]. However, in RIPF, the role of irradiated endothelial cells undergoing EndMT in the activation of fibroblasts into myofibroblasts and the underlying mechanism have not been reported.

Taken together, we propose the hypothesis that irradiated endothelial cells undergo Snail-induced EndMT to promote the differentiation of fibroblasts into myofibroblasts. Therefore, in the present study we investigated in vitro whether and how Snail-induced EndMT contributes to myofibroblast activation, which could serve as a target for the prevention and treatment of RIPF.

## 2. Materials and Methods

### 2.1. Cells and Irradiation

Human fetal lung fibroblasts (MRC-5) and human umbilical vein endothelial cells (HUVECs) (Cell Bank, Chinese Academy of Sciences, China) were, respectively, cultured in minimal essential media (MEM) and Dulbecco's minimal essential media (DMEM) supplemented with 10% fetal bovine serum (FBS, Gibco) and were maintained in a humidified incubator with 5% CO_2_ at 37°C. Cells were irradiated using a X-ray irradiator (RS 2000 Biological System irradiator, Rad Source, USA).

### 2.2. Coculture and Cell Sorting by Flow Cytometry

Noncontact cells were cocultured in six-well plates with a 0.4 *μ*m pore polyester membrane insert (Corning). Contact cells were cocultured in 25-cm^2^ cell culture flasks (Corning). MRC-5 cells were labeled with green fluorescent protein (GFP) and cocultured with HUVECs at a ratio of 9 : 1. After coculture, cells were separated by flow cytometry according to fluorescence.

### 2.3. RNA Extraction and Real-Time Quantitative PCR

Cells were lysed in RNAiso Plus (Takara) for total RNA extraction. The quantity and quality of RNAs were examined using NanoDrop™ 2000 c spectrophotometers, and 500 ng of RNA of each sample was subjected to reverse transcription to complementary DNA (cDNA) using the RevertAid First-Strand cDNA Synthesis Kit (Thermo Scientific). Quantitative PCR was performed on a 7900 HT Fast Real-Time PCR System with results normalized to the *β*-actin. The ΔΔCT method was used to calculate relative expression. Primer sequences used in quantitative PCR are shown in Supplementary Table S1.

For miRNA expression analysis, RNA was reverse transcribed using a miDETECT A Track miRNA qRT-PCR Kit and a Bulge-Loop miRNA qRT-PCR Kit (RiboBio) according to the manufacturer's instructions. Expression levels were normalized to U6-snRNA expression. All experiments were performed in triplicate.

### 2.4. Oligonucleotide Construction and Transfection

Mimics and inhibitors of miRNA and negative control oligonucleotides for hsa-miR-199a-5p were obtained from RiboBio. Target cells were transfected with oligonucleotides using Lipofectamine 2000 reagent (Invitrogen) according to the manufacturer's instructions. MRC-5 were infected with lentivirus expressing GFP. HUVECs were infected with lentivirus expressing Snail. Lentivirus for Snail and GFP were both purchased from GeneChem.

### 2.5. Western Blot Analysis

Protein was extracted in RIPA lysis and extraction buffer and cleared by centrifugation. Protein extracts were separated using SDS-PAGE, transferred to a 0.45 *μ*m Immobilon-P PVDF membrane (Millipore), and immunoblotted using the indicated antibodies followed by SuperSignal West Pico Chemiluminescent Substrate (Thermo Scientific). Western blotting was performed using the following antibodies: Snail, 1 : 1000 (Cell Signaling Technology); CD31, 1 : 1000 (Abclonal); vimentin, 1 : 1000 (Cell Signaling Technology); *α*-SMA, 1 : 200 (Boster); collagen type I, 1 : 200 (Boster); and *β*-actin 1 : 1000 (Boster). The blots were analyzed using G: BOX Chemi X system (Syngene).

### 2.6. Immunofluorescence Assay

Cells were fixed in 4% paraformaldehyde and permeabilized with 0.5% Triton in PBS. Cells were incubated with anti-*α*-SMA antibody at a concentration recommended by the manufacturer at 25°C for 1 hour and subsequently incubated with rhodamine-labeled secondary antibody. To stain stress fibers, cells were incubated with fluorescein isothiocyanate -conjugated phalloidin at 25°C for 1 hour. Nuclei were counterstained with DAPI and images were taken using a fluorescence microscopy.

### 2.7. Transwell Migration Assay

5 × 10^4^ HUVECs in 200 *μ*L DMEM were seeded onto the top chamber of the 24-well with an 8 *μ*m pore polyester membrane (Corning) while the bottom chamber was filled with 500 ul DMEM with 10% FBS. After 24 hours, cells that crossed the membranes were fixed with 95% methanol, stained with 0.1% crystal violet, and observed under optical microscope.

### 2.8. Tube Formation Assay

100 *μ*L matrigel was pipetted into each well of a 48-well plate and polymerized for 45 minutes at 37°C. HUVECs (1 × 10^4^) in 200 *μ*L DMEM were added to each well and incubated at 37°C in 5% CO_2_ for 6 hours. Pictures were taken under a 100x bright-field microscope.

### 2.9. Statistical Analysis

Statistical calculations were performed using GraphPad Prism 6 software. Statistical comparisons between experimental groups were analyzed by two-tailed Student's *t*-test. Data are presented as mean ± SEM. *P* < 0.05 was considered significant.

## 3. Results

### 3.1. Irradiated Endothelial Cells Promoted Myofibroblast Differentiation

A key feature of myofibroblasts is the expression of alpha-smooth muscle actin (*α*-SMA), along with elevated expression of collagen type I. To determine whether MRC-5 were activated from fibroblasts into myofibroblasts, we defined *α*-SMA and collagen type I as biomarkers of MRC-5 activation. Cells were exposed to 0, 2, 4, 6, 8, and 10 Gy of X-ray radiation. With a radiation dosage of 8 Gy, cells showed higher levels of *α*-SMA and collagen expression (Supplementary Fig.  S1). Thus, we chose 8 Gy as radiation dose in the following experiments.

**Figure 1:**
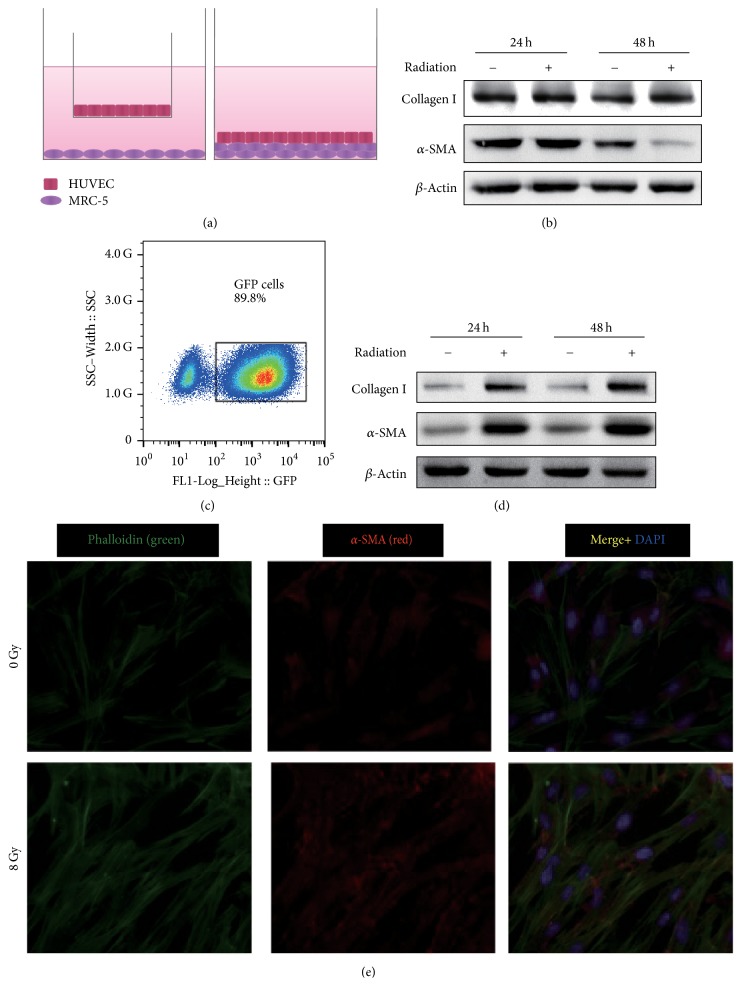
Irradiated HUVECs promote the expression of MRC-5 fibrotic markers. Panel (a): scheme illustrating the procedure to evaluate the contribution of HUVECs to MRC-5 fibrotic markers. MRC-5 cells were cocultured with irradiated HUVECs with no physical contact (noncontact). MRC-5 cells were cocultured with irradiated HUVECs with physical contact (contact). Panel (b): HUVECs were preincubated in the presence or absence of irradiation and then coincubated with MRC-5 cells (noncontact). After incubation for 24 or 48 h, the MRC-5 cells were harvested. Total protein was extracted. Western blot shows the protein expression of collagen I and *α*-SMA. Panel (c): cell sorting by flow cytometry. MRC-5 cells were labeled with fluorescent GFP (green cells) and plated with unlabeled HUVECs at a ratio of 9 : 1. Panel (d): HUVECs were preincubated in the presence or absence of radiation and then coincubated with MRC-5 cells (contact). After incubation for 24 or 48 h, the MRC-5 cells were sorted by flow cytometry and harvested. Western blot was performed to examine total protein. Panel (e): dual immunofluorescence analysis of *α*-SMA and stress fibers stained with phalloidin green. Colocalization of stress fibers and *α*-SMA in MRC-5 was evident under treatment of irradiated HUVECs.

We established two different coculture systems to evaluate the effect of irradiated HUVECs on MRC-5 activation (Figure 1(a)). By using the noncontact coculture system, irradiated HUVECs did not affect the expression of* COL1A1* (collagen type I alpha 1 chain) or* ACTA2* (*α*-SMA) in MRC-5 cells (Figure 1(b), Fig.  S2A). To determine whether irradiated HUVECs affected MRC-5 activation in the contact coculture system, HUVECs and GFP-labeled MRC-5 were cocultured at a ratio of 1 : 9. After 24 or 48 h, cells were sorted by flow cytometry according to the fluorescence (Figure 1(c)). At the protein level, both collagen and *α*-SMA were significantly elevated in MRC-5 cells treated with irradiated HUVECs (Figure 1(d)). The same results were observed at 48 h at the mRNA level (Fig.  S2B). Therefore, we chose the contact coculture system in the following experiments.

To illustrate phenotypical changes during fibroblast-myofibroblast differentiation, dual immunofluorescence analysis of *α*-SMA and stress fibers stained with phalloidin green was conducted. In the contact coculture system, colocalization of stress fibers and *α*-SMA in MRC-5 was evident under treatment of irradiated HUVECs (Figure 1(e)).

### 3.2. Irradiated HUVECs Underwent Snail-Induced EndMT

**Figure 2:**
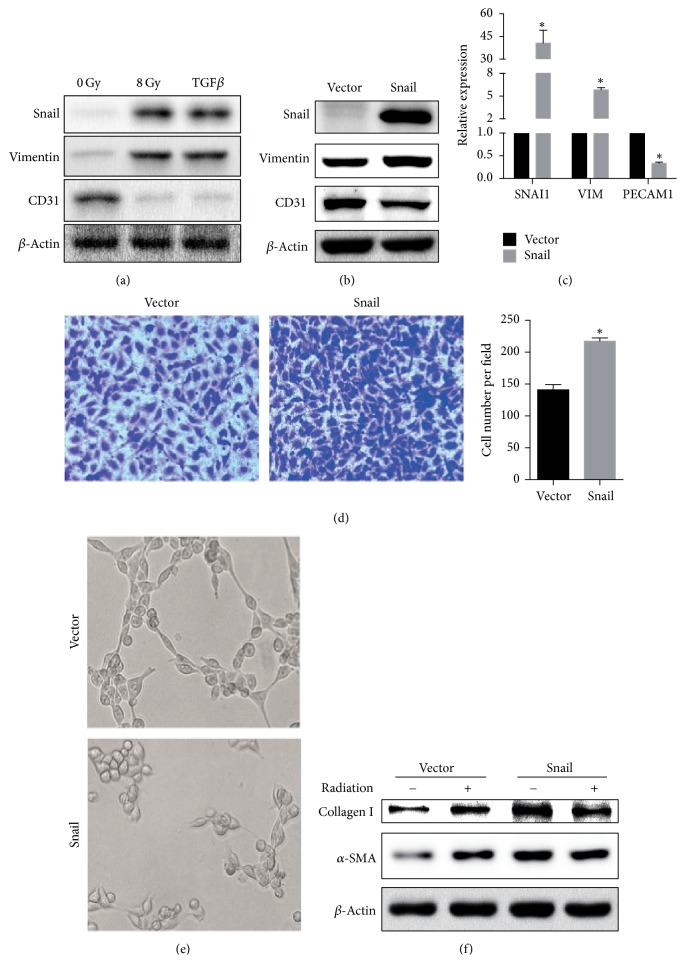
Radiation induced EndMT contributes to the fibrotic effect in MRC-5. Panel (a): HUVECs were incubated in the presence or absence of irradiation. After incubation for 48 h, cells were harvested. The expression of Snail, vimentin, and CD31 was examined. Panels (b) and (c): Snail overexpression reduced the expression of CD31, while increasing the expression of vimentin. Panel (d): Snail overexpression in HUVECs significantly increased migration capacity. Panel (e): Snail overexpression led to impaired ability of endothelial cells to form capillary-like structures. Panel (f): Snail-overexpressed HUVECs were preincubated in the presence or absence of irradiation and then coincubated with MRC-5 (contact). After incubation for 48 h, cells were sorted by flow cytometry and MRC-5 were harvested. Total protein was examined by western blot. Error bars represent SEM from three replicates (^*∗*^*P* < 0.05).

To investigate the changes in endothelial cells after radiation, HUVECs were treated with 8 Gy of radiation or stimulated with TGF*β* which was a potent inducer of Snail as positive control. We chose the time point of 48 h for observation (Fig.  S3A). After 48 h, both Snail and vimentin expression in TGF*β* treated and irradiated endothelial cells were much higher than that in the control group. We also found that the expression of endothelial cell marker CD31 was significantly decreased after irradiation (Figure 2(a)).

### 3.3. Snail Overexpression Induced an EndMT-Like Process in HUVECs

We examined whether Snail overexpression in HUVECs caused EndMT in a manner similar to radiation. During EndMT, endothelial cells lose their endothelial characteristics and intercellular adhesion while acquiring mesenchymal properties. Snail overexpression in HUVECs reduced the expression of the endothelial cell marker CD31 and increased the expression of the mesenchymal cell marker vimentin (Figure 2(b)). The same results were observed at the mRNA level (Figure 2(c)).

Similar to EndMT properties, Snail overexpression in HUVECs accompanied significantly increased migration capacity (Figure 2(d)). Subsequently, we used a matrigel tube formation assay to explore if Snail could affect the ability of HUVECs to form capillary-like structures. Snail overexpression led to impaired ability of endothelial cells to form capillary-like structures (Figure 2(e)). Cell viability was determined by CCK8 assay and there was no statistical difference between two groups (data not shown).

### 3.4. Snail Overexpression in HUVECs Promoted Myofibroblast Differentiation

To verify that Snail-induced EndMT played a role in myofibroblast differentiation, we cocultured MRC-5 with Snail-overexpressed HUVECs for 48 h. Upregulation of collagen and *α*-SMA was found in the Snail overexpression group. The results were consistent with those of the radiation group. However, when MRC-5 cells were cocultured with HUVECs, which were both Snail-overexpressed and irradiated, the expression of fibrotic markers in MRC-5 did not increase compared to the group that was only irradiated. When MRC-5 cells were cocultured with Snail-overexpressed HUVECs, the expression levels of *α*-SMA and collagen type I were not significantly different between the irradiated group and control group (Figure 2(f), Fig.  S3B).

### 3.5. Snail Increased miR-199a-5p Expression in HUVECs

**Figure 3:**
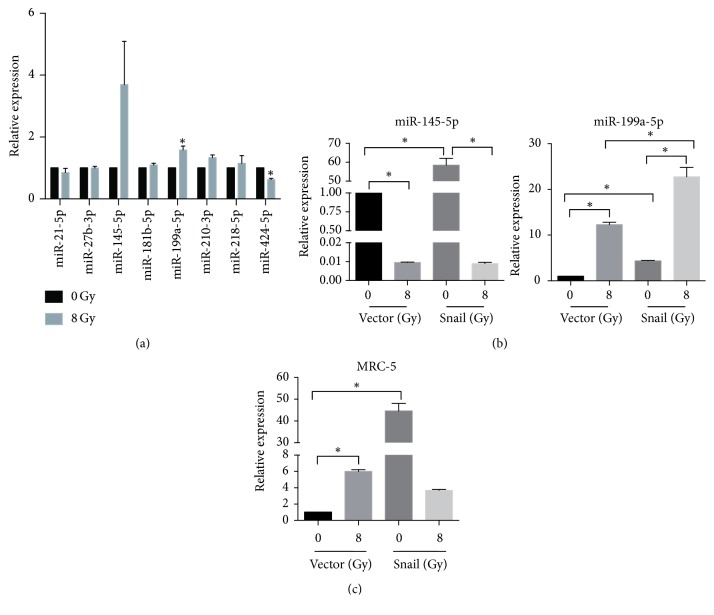
Snail-induced miR-199a-5p promotes the expression of MRC-5 fibrotic markers. Panel (a): eight fibrosis-associated microRNAs were selected. The expression levels of miR-145-5p and miR-199a-5p were increased in irradiated HUVECs compared to that in the control group. Panel (b): the expression of miR-145-5p and miR-199a-5p in Snail-overexpressed HUVECs. Panel (c): transfer of miR-199a-5p from HUVECs to cocultured MRC-5 cells. Total RNA was extracted. The miRNA expression was measured using real-time qPCR. Error bars represent SEM from three replicates (^*∗*^*P* < 0.05).

Snail-overexpressed HUVECs induced the transition of fibroblasts into myofibroblasts, suggesting that some fibrosis-stimulating substances might be transferred from EndMT-like cells to fibroblasts. We investigated several key miRNAs that are known to promote the differentiation of fibroblasts into myofibroblasts: miR-21-5p [14], miR-27b-3p [15], miR-145-5p [16], miR-181b-5p [17], miR-199a-5p [18], miR-210-3p [19], miR-218-5p [20], and miR-424-5p [21]. After radiation, the expression of miR-145-5p and miR-199a-5p was upregulated in HUVECs (Figure 3(a)). Only miR-199a-5p expression was increased by Snail overexpression (Figure 3(b)). Furthermore, both Snail-overexpressed and irradiated HUVECs showed increased miR-199a-5p expression in cocultured MRC-5 cells (Figure 3(c)).

### 3.6. Snail-Induced miR-199a-5p Elevation Promoted Myofibroblast Differentiation

**Figure 4:**
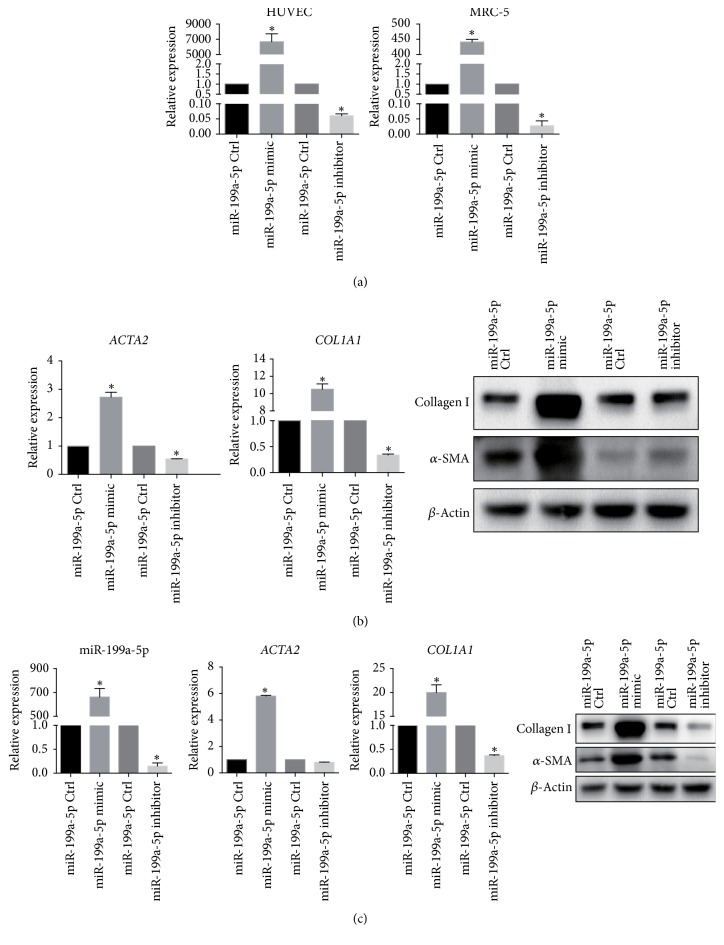
Transfer of miR-199a-5p from HUVECs to cocultured MRC-5 cells promotes the expression of MRC-5 fibrotic markers. Panel (a): HUVECs were transfected with miR-199a-5p mimic, miR-199a-5p inhibitor, and control vector. miR-199a-5p was transferred from HUVECs to MRC-5. Panel (b): miR-199a-5p was transferred from HUVECs to MRC-5 cells and promoted the expression of collagen I and *α*-SMA in MRC-5 cells. Panel (c): MRC-5 cells were transfected with miR-199a-5p mimic, miR-199a-5p inhibitor, and control vector. Fibrotic markers were examined after such transfection. Error bars represent SEM from three replicates (^*∗*^*P* < 0.05).

To investigate whether elevated miR-199a-5p in HUVECs affected the differentiation of fibroblasts into myofibroblasts, we transfected HUVECs with miR-199a-5p mimic or inhibitor. Then, we cultured the cells with GFP-labeled MRC-5. After 48 h of coculture, the cell types were sorted by flow cytometry and the miR-199-5p level was determined in each population. Both HUVECs and MRC-5 cells expressed high levels of miR-199a-5p when transfected with miR-199a-5p mimic. Furthermore, reduced levels of miR-199a-5p were detected when HUVECs were transfected with miR-199a-5p inhibitor (Figure 4(a)). We subsequently tested the fibrotic markers of cocultured MRC-5 cells and found that both *α*-SMA and collagen type I expression were upregulated after transfecting HUVECs with miR-199a-5p mimic. Although a significant decrease in* ACTA2* and* COL1A1* mRNA expression was observed in MRC-5 cells, we failed to detect any decrease in either *α*-SMA or collagen type I at the protein level (Figure 4(b)). To verify the direct effect of miR-199a-5p on fibroblasts, we transfected MRC-5 cells with miR-199a-5p mimic or inhibitor. As expected, such transfection affected the *α*-SMA and collagen type I expression in MRC-5 as expected (Figure 4(c)). The same results were observed at the mRNA level.

## 4. Discussion

The pathogenesis of RIPF remains controversial; however, fibroblast activation has been reported as a major factor in fibrosis [22]. The basic biology of fibrosis is the activation or differentiation of fibroblasts into myofibroblasts, which abundantly express *α*-SMA, produce collagen, and induce ECM deposition [23]. In addition, myofibroblasts may also be derived from EndMT, and studies have shown that EndMT plays an important role in RIPF [10]. To our knowledge, the present study is the first to report that irradiated endothelial cells undergo EndMT via the Snail/miR-199a-5p axis to promote the differentiation of fibroblasts into myofibroblasts.

Since fibrosis usually occurs around the blood vessels at first [24] and fibroblasts and endothelial cells were both vital in fibrosis, we proposed the concept of endothelial cells and fibroblasts cross-talk in RIPF. We have demonstrated that EndMT-like changes in irradiated endothelial cells promoted the activation of fibroblasts into myofibroblasts. In addition, nonirradiated endothelial cells had no effect on myofibroblast differentiation. Interestingly, this effect occurred only when the irradiated endothelial cells and fibroblasts were cultured with physical contact. This suggested that endothelial cells did not interact with fibroblasts in a paracrine manner and that endothelial cells and fibroblasts might interact with each other through intercellular physical contact [25]. These data indicate the importance of cross-talk between endothelial cells and fibroblasts in RIPF.

Snail is an important regulator of renal fibrosis [26], cardiac fibrosis [27], and pulmonary fibrosis [28] and is known for its ability to trigger epithelial-mesenchymal transition (EMT) and EndMT [29]. Studies have reported that radiation can activate lung epithelial cells, resulting in Snail-induced EMT [30]. In renal fibrosis, renal tubular epithelial cell damage promotes Snail-induced partial EMT, in which epithelial cells do not directly differentiate into myofibroblasts but transduce signals to interstitial cells to promote fibroblast differentiation into myofibroblasts [31]. Since EndMT is a special form of EMT [32], we speculated that endothelial cells can undergo a partial EndMT process as well. In cardiac fibrosis, a Snail-induced EndMT-like process in endothelial cells promoted myofibroblast activation [13]. In our study, we proposed that irradiated endothelial cells processed Snail-induced partial EndMT to promote the activation of fibroblasts into myofibroblasts. Partial EndMT resulted in partial loss of the endothelial marker CD31, whereas collagen synthesis capacity was not yet obtained. At this time, the endothelial cells did not directly differentiate into activated myofibroblasts but produced some fibrotic factors acting on adjacent fibroblasts.

We found that miR-199a-5p was profibrotic mediator downstream of Snail. miR-199a-5p has been shown to play an important role in a variety of organ fibrosis [18, 33, 34], and we have demonstrated, for the first time, its status in Snail-induced EndMT in RIPF. When coculturing irradiated or Snail-overexpressed HUVECs with fibroblasts, it was found that in addition to increased expression of miR-199a-5p in endothelial cells, miR-199a-5p expression in fibroblasts was also unexpectedly increased, suggesting that miR-199a-5p is located downstream of Snail-induced EndMT and that it could be transferred from endothelial cells to fibroblasts.

To investigate whether miR-199a-5p had an impact on fibroblasts, we transfected HUVECs with miR-199a-5p mimic or inhibitor and examined the effects in both HUVECs and MRC-5 cells. While fibroblasts were activated as myofibroblasts, there was no significant change in fibrotic marker in endothelial cells (data not shown). Then, we transfected MRC-5 with miR-199a-5p mimic or inhibitor, and the expression of the fibrotic marker in fibroblasts was altered accordingly. It has been reported that miR-199a-5p promotes the differentiation of fibroblasts into myofibroblasts by regulating caveolin-1, a key mediator of pulmonary fibrosis, to induce pulmonary fibrosis [18]. Therefore, miR-199a-5p may promote the activation of fibroblasts by downregulating caveolin-1 in fibroblasts in radiation induced microenvironment cross-talk.

Endothelial cells, alveolar epithelial cells, macrophages, and fibroblasts are involved in the occurrence of RIPF, during which they interact and constitute the microenvironment of RIPF [35]. Myofibroblasts are the core cells of RIPF, which produce collagen and other matrix molecules. Cells secrete active ingredients to promote the formation of myofibroblasts, some of which can directly transform into myofibroblasts. All of these cells interact with each other through inflammatory responses, paracrine effects, oxidative stress, and other signaling pathways. However, the specific mechanism is still controversial. The current treatments for RIPF include amifostine (to reduce oxidative damage), glucocorticosteroids (to restore the immunological balance), and other symptomatic and supportive treatments [36]; however, the effects of these treatments are limited. The present study is the first to propose that irradiated endothelial cells undergoing EndMT promoted myofibroblast activation via the Snail/miR-199a-5p axis, providing new targets for the prevention and treatment of RIPF, consequently enhancing the effect of radiotherapy for non-small-cell lung cancer. Our study provided a more precise way to treat pulmonary fibrosis, which targeted protein molecules in a specific cell type. Precision treatment like that minimizes the adverse impacts on the function of other normal cells, thus reducing side effects, as compared to current approaches. In further investigation, we will establish Snail/miR-199a-5p conditional knockout mouse, in which miR-199a-5p inactivation is restricted to the endothelial cells to confirm current results.

In conclusion, our experiments showed that irradiated endothelial cells undergoing EndMT promoted myofibroblast activation via the Snail/miR-199a-5p axis. Inhibition of Snail or miR-199a-5p in endothelial cells could be a promising strategy for the prevention or treatment of RIPF.

## Supplementary Materials

Supplementary Materials Fig. S1: cells were exposed to 0, 2, 4, 6, 8, and 10 Gy of X-ray radiation. Fig. S2: real-time qPCR analysis of irradiated HUVECs promoting the expression of MRC-5 fibrotic markers. Panel A: HUVECs were preincubated in the presence or absence of irradiation and then coincubated with MRC-5 cells (noncontact). After incubation for 24 or 48 h, the MRC-5 cells were harvested. Total RNA was extracted. Real-time qPCR shows the transcriptional expression of COL1A1 and ACTA2. Panel B: HUVECs were preincubated in the presence or absence of radiation and then coincubated with MRC-5 cells (contact). After incubation for 24 or 48 h, the MRC-5 cells were sorted by flow cytometry and harvested. Real-time qPCR was performed to examine total RNA. Error bars represent SEM from three replicates (^*∗*^*P* < 0.05). Fig. S3: real-time qPCR analysis of radiation induced EndMT contributes to the fibrotic effect in MRC-5. Panel A: HUVECs were incubated in the presence or absence of irradiation. After incubation for 24 or 48 h, cells were harvested. Total RNA and protein were extracted. The expression of Snail and CD31 was examined. Panel B: HUVECs overexpressing Snail were preincubated in the presence or absence of irradiation and then coincubated with MRC-5 (contact). After incubation for 48 h, cells were sorted by flow cytometry and MRC-5 were harvested. Total RNA was extracted and examined. Table S1: primers for qPCR.
