# Supplementary material for: Irradiated Human Umbilical Vein Endothelial Cells Undergo Endothelial-Mesenchymal Transition via the Snail/miR-199a-5p Axis to Promote the Differentiation of Fibroblasts into Myofibroblasts
Source: Biomed Res Int. 2018 Feb 12;2018:4135806. doi: 10.1155/2018/4135806 (PMC5830288; doi:10.1155/2018/4135806)
Supplement: Supplementary Materials — Fig. S1: cells were exposed to 0, 2, 4, 6, 8, and 10 Gy of X-ray radiation. Fig. S2: real-time qPCR analysis of irradiated HUVECs promoting the expression of MRC-5 fibrotic markers. Panel A: HUVECs were preincubated in the presence or absence of irradiation and then coincubated with MRC-5 cells (noncontact). After incubation for 24 or 48 h, the MRC-5 cells were harvested. Total RNA was extracted. Real-time qPCR shows the transcriptional expression of COL1A1 and ACTA2. Panel B: HUVECs were preincubated in the presence or absence of radiation and then coincubated with MRC-5 cells (contact). After incubation for 24 or 48 h, the MRC-5 cells were sorted by flow cytometry and harvested. Real-time qPCR was performed to examine total RNA. Error bars represent SEM from three replicates (∗P < 0.05). Fig. S3: real-time qPCR analysis of radiation induced EndMT contributes to the fibrotic effect in MRC-5. Panel A: HUVECs were incubated in the presence or absence of irradiation. After incubation for 24 or 48 h, cells were harvested. Total RNA and protein were extracted. The expression of Snail and CD31 was examined. Panel B: HUVECs overexpressing Snail were preincubated in the presence or absence of irradiation and then coincubated with MRC-5 (contact). After incubation for 48 h, cells were sorted by flow cytometry and MRC-5 were harvested. Total RNA was extracted and examined. Table S1: primers for qPCR. [file 4135806.f1.pdf]

Figure S1

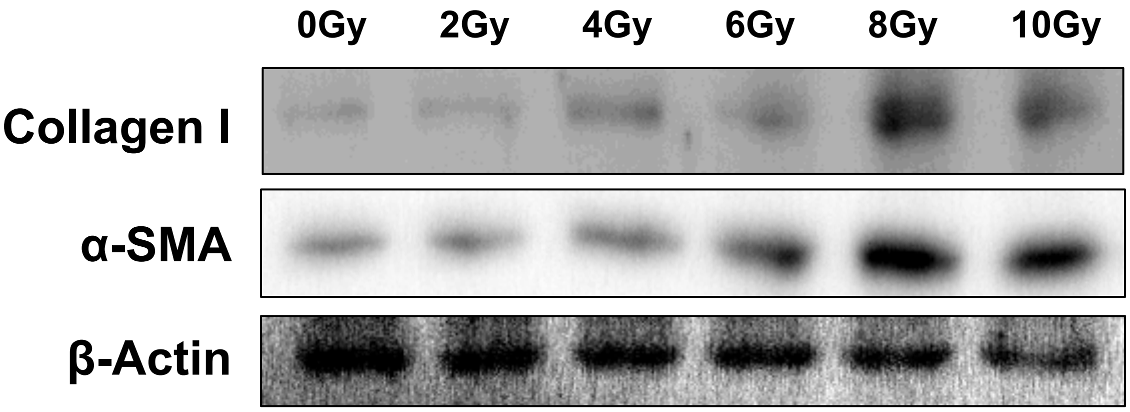

Fig S1. Cells were exposed to 0, 2, 4, 6, 8, 10 Gy of x-ray radiation.

Figure S2

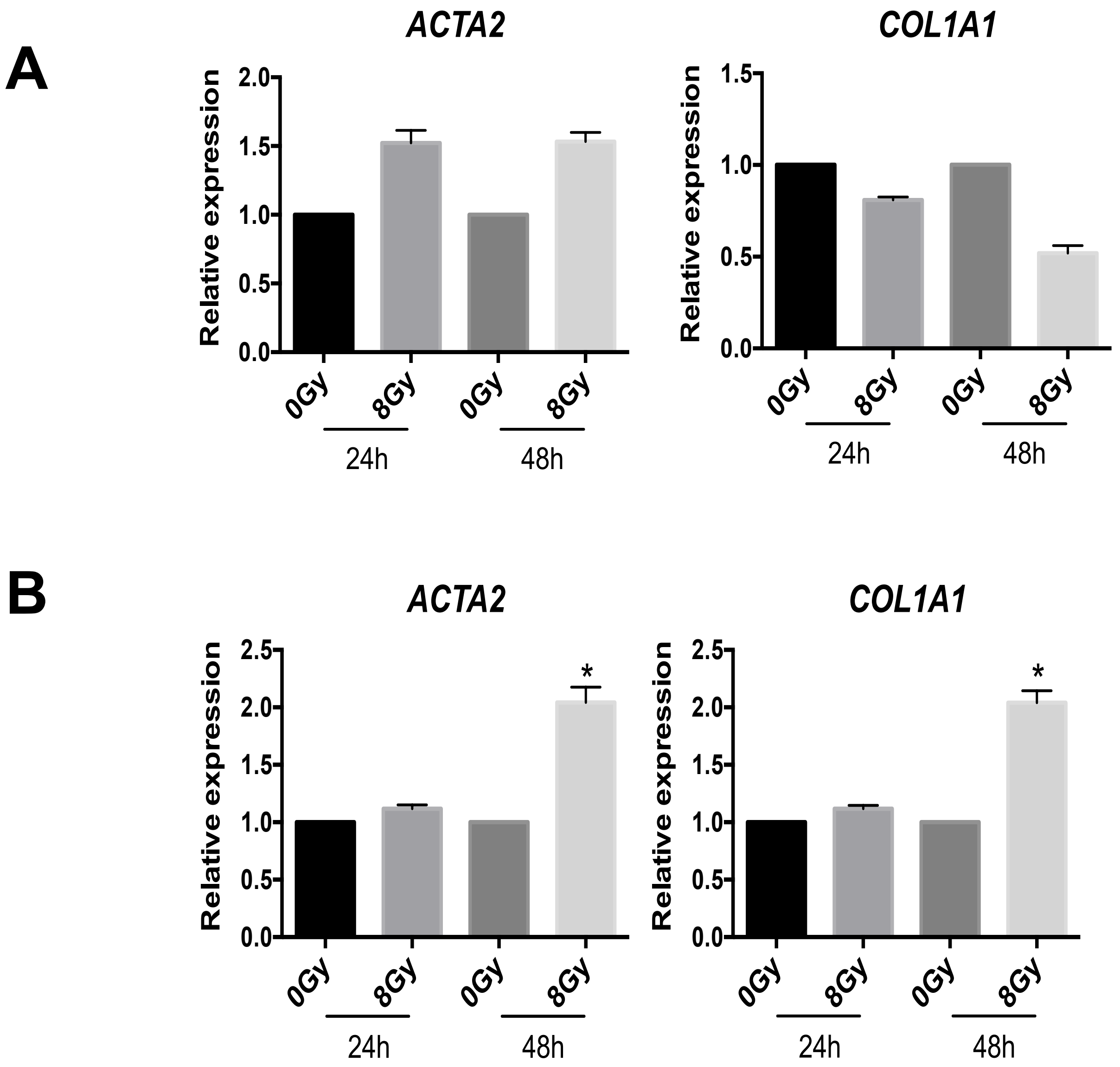

Fig S2. Panel A: HUVECs were pre-incubated in the presence or absence of irradiation and then co-incubated with MRC-5 cells (non-contact). After incubation for 24 or 48 h, the MRC-5 cells were harvested. Total RNA were extracted. Real-time qPCR shows the transcriptional expression of *COL1A1* and *ACTA2*. Panel B: HUVECs were pre-incubated in the presence or absence of radiation and then co-incubated with MRC-5 cells (contact). After incubation for 24 or 48 h, the MRC-5 cells were sorted by flow cytometry and harvested. Real-time qPCR was performed to examine total RNA. Error bars represent SEM from three replicates (*P<0.05).

Figure S3

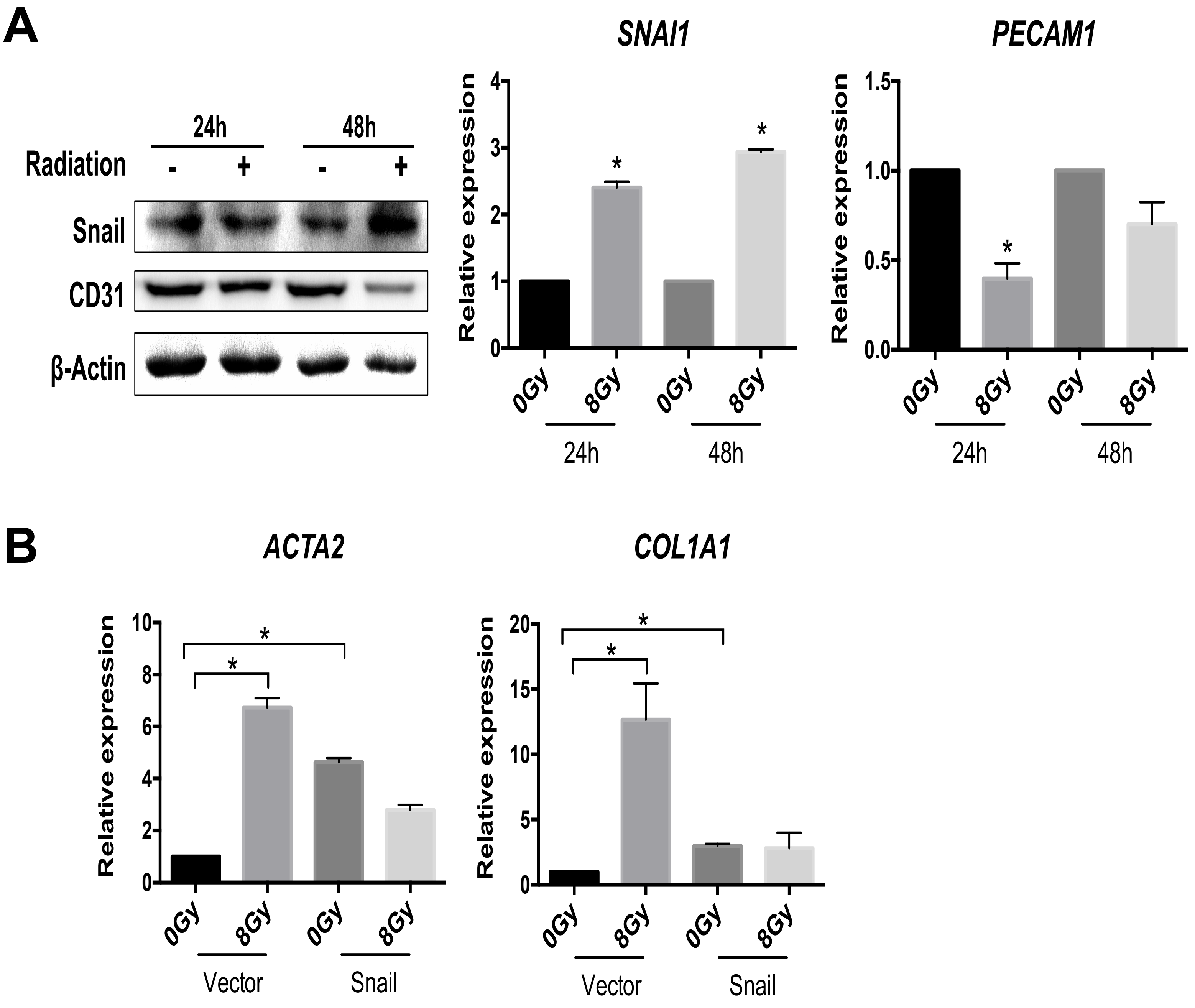

Fig S3. Panel A: HUVECs were incubated in the presence or absence of irradiation. After incubation for 24 or 48 h, cells were harvested. Total RNA and protein were extracted. The expression of Snail and CD31 were examined. Panel B: HUVECs overexpressing Snail were pre-incubated in the presence or absence of irradiation and then co-incubated with MRC-5 (contact). After incubation for 48 h, cells were sorted by flow cytometry and MRC-5 were harvested. Total RNA was extracted and examined.

Table S1. PCR primers for quantitative PCR.

	Forward Primer	Reverse Primer
ACTA2	AAAAGACAGCTACGTGGGTGA	GCCATGTTCTATCGGGTACTTC
COL1A1	GAGGGCCAAGACGAAGACATC	CAGATCACGTCATCGCACAAC
SNAI1	TCGGAAGCCTAACTACAGCGA	AGATGAGCATTGGCAGCGAG
PECAM1	AACAGTGTTGACATGAAGAGCC	TGTAAAACAGCACGTCATCCTT
ACTB	CATGTACGTTGCTATCCAGGC	CTCCTTAATGTCACGCACGAT
